# Systematic review of education and practical guidance on regression modeling for medical researchers who lack a strong statistical background: Study protocol

**DOI:** 10.1371/journal.pone.0241427

**Published:** 2020-12-21

**Authors:** Paul Bach, Christine Wallisch, Nadja Klein, Lorena Hafermann, Willi Sauerbrei, Ewout W. Steyerberg, Georg Heinze, Geraldine Rauch, for topic group 2 of the STRATOS initiative

**Affiliations:** 1 Corporate Member of Freie Universität Berlin, Humboldt-Universität zu Berlin, and Berlin Institute of Health, Institute of Biometry and Clinical Epidemiology, Charité - Universitätsmedizin Berlin, Berlin, Germany; 2 Berlin Institute of Health (BIH), Berlin, Germany; 3 School of Business and Economics, Applied Statistics, Humboldt-Universität zu Berlin, Berlin, Germany; 4 Section for Clinical Biometrics, Center for Medical Statistics, Informatics and Intelligent Systems, Medical University of Vienna, Vienna, Austria; 5 Institute of Medical Biometry and Statistics, Faculty of Medicine and Medical Center—University of Freiburg, Freiburg, Germany; 6 Department of Biomedical Data Sciences, Leiden University Medical Center, Leiden, The Netherlands; Institute for Quality and Efficienty in Health Care (IQWiG), GERMANY

## Abstract

In the last decades, statistical methodology has developed rapidly, in particular in the field of regression modeling. Multivariable regression models are applied in almost all medical research projects. Therefore, the potential impact of statistical misconceptions within this field can be enormous Indeed, the current theoretical statistical knowledge is not always adequately transferred to the current practice in medical statistics. Some medical journals have identified this problem and published isolated statistical articles and even whole series thereof. In this systematic review, we aim to assess the current level of education on regression modeling that is provided to medical researchers via series of statistical articles published in medical journals. The present manuscript is a protocol for a systematic review that aims to assess which aspects of regression modeling are covered by statistical series published in medical journals that intend to train and guide applied medical researchers with limited statistical knowledge. Statistical paper series cannot easily be summarized and identified by common keywords in an electronic search engine like Scopus. We therefore identified series by a systematic request to statistical experts who are part or related to the STRATOS Initiative (STRengthening Analytical Thinking for Observational Studies). Within each identified article, two raters will independently check the content of the articles with respect to a predefined list of key aspects related to regression modeling. The content analysis of the topic-relevant articles will be performed using a predefined report form to assess the content as objectively as possible. Any disputes will be resolved by a third reviewer. Summary analyses will identify potential methodological gaps and misconceptions that may have an important impact on the quality of analyses in medical research. This review will thus provide a basis for future guidance papers and tutorials in the field of regression modeling which will enable medical researchers 1) to interpret publications in a correct way, 2) to perform basic statistical analyses in a correct way and 3) to identify situations when the help of a statistical expert is required.

## Introduction

### Rationale

In the last decades, intensive global research activities led to a huge general medical progress. Likewise, biostatistical methodology has developed rapidly. However, the developments in both fields did not move forward hand in hand and indeed, the available methodological knowledge has not yet been fully integrated in medical publications. One reason is that many statistical analyses are not conducted by professional experts but by researchers with limited statistical background to reduce time or financial resources or because a professional statistician is not available. However, such researchers cannot be aware of all statistical pitfalls and will usually not overview the latest developments in statistical methodology, which is already a challenge for a professional biostatistician. Appropriate guidance and tutorials are often missing for medical researchers with a limited background in statistical methodology as the available statistical articles are often written in a rather technical manner.

Some medical journals have identified this problem and, as a consequence, publish isolated statistical articles or even whole series of statistical articles with a tutorial character. These articles play a key role for the statistical education of medical researchers as they introduce new methods and point out possible misconceptions in less technical and mathematical detail. Moreover, the observed problem of a limited integration of statistical research within medical publications has led to the formation of the STRATOS Initiative, which is a collaborative network of experts with background in many different areas of biostatistical and epidemiological methods interest (see Table 1 in ref [1]). The long-term aim of the initiative is to provide accessible and evidence-based guidance on key topics in the design and analysis of observational studies. Thereby, different audience groups are addressed varying in their level of statistical education, experience and interest. In particular, this includes medical researchers with limited statistical knowledge who presumably conduct the majority of statistical analyses.

While the gap between the existing knowledge and current practice is apparent in almost all fields of medical statistics, this work focuses on the specific topic of multivariable regression modeling. Some common aims of regression modeling are 1) to adjust for confounders when estimating effects of interventions or exposures, 2) to identify influential variables associated with specific disease outcomes and 3) to predict specific disease outcomes [2]. The most common regression models are the linear, the logistic, the Cox and the Poisson regression model to analyze continuous, binary, time-to-event or count data, respectively.

Multivariable regression models are applied in almost all medical research projects, especially in the context of observational studies. One problem however is that there is no global state of the art methodology which applies to all types of data situations [3]. Still, there is a broad agreement among experts on many misconceptions, which have been discussed in the statistical literature for decades but they are still prevalent in medical research publications. The impact of such statistical misconceptions within this field can be enormous. For example, data-driven variable selection techniques are frequently applied being unaware of associated issues such as bias in post-selection p-values and inference, as well as instability of the selection [4, 5]. Furthermore, fundamentally flawed techniques like univariate screening are still often applied for variable selection [6, 7]. In contrast, more advanced selection techniques like the Lasso or Bayesian variable selection techniques are hardly ever used for the analysis of low-dimensional data [8, 9]. Another common misconception is that continuous predictors are typically either dichotomized by rather arbitrary cut-points, leading to bias, loss of information and power, or it is taken for granted that they have a linear effect on the outcome of interest [10, 11]. Flexible solutions like fractional polynomials, splines and generalized additive models would avoid those undesirable consequences and could provide further clinical insights but are rarely ever used in practice [11–15]. These misconceptions related to regression models are exemplary illustrations of the current problems. There exist many other related topics, which are also highly relevant for the interpretation of medical research results but are beyond the scope of this review. We will exclusively focus on statistical series in medical journals. It has to be acknowledged that there are other excellent sources of education and guidance for medical researchers such as video tutorials, workshops and textbook, but we will not consider them here as a fair comparison of these different formats is difficult.

### Objectives

The global objective of this review is to provide an evidence basis for future guidance papers and tutorials in the field of regression modeling which will enable medical researchers 1) to interpret publications in a correct way, 2) to perform basic statistical analyses in an adequate way and 3) to identify situations when the help of a statistical expert is required. This is the protocol for a systematic review that aims to assess which aspects of regression modeling are covered by statistical series published in medical journals that intend to train and guide applied medical researchers with limited statistical knowledge. Within the identified series, we will search for topic-relevant articles on regression modeling. Subsequently, we will systematically check the content of these articles with respect to a predefined list of statistical aspects related to regression modeling. Thereby, the following general questions will be assessed:

- Which type of regression models and which general aspects of regression modeling are discussed?
- Which aspects regarding interpretation of regression models are considered?
- Which methods of data-driven variable selection are discussed, recommended or discouraged from?
- Which type of guidance is given on sample size requirements?
- What kind of software recommendations are given?

To perform the series screening and the content analysis of the topic-relevant articles, we will use predefined report forms. Using these, we will assess whether aspects related to the questions above are discussed and if so, in which intensity. Moreover, we will assess if clear recommendations or warnings against suboptimal strategies are issued. Furthermore, we will check whether illustrative examples and software commands are provided enabling the applied medical researcher to succeed on her/his own. The report forms will be filled out by two independent raters to ensure a high level of reliability. The objectives and the design of the planned study were discussed with several medical researchers from the target audience.

## Material and methods

### Eligibility criteria

This is a comprehensive review to identify topic-relevant articles on regression modeling within statistical series published in medical journals, which are defined as journals with a target audience mainly or exclusively consisting of medical researchers or practitioners. With the expression “medical researcher” we refer to any researcher with completed studies in medicine or equivalent education, whereas “practitioner” refers to qualified medical experts not necessarily being active in research.

Note that there might be journals with an epidemiological focus, where the target audience is not homogeneous. If the target audience of a journal consists of medical experts and epidemiologists, we conservatively will include the journal. Journals are thus only excluded if the target audience has a pure theoretical, methodological or statistical focus. We will assess all medical journals as potentially eligible if they are available in English language, as English publications have a broader visibility and an international impact. Moreover, we consider medical journals in which several articles with a statistical focus have been published in the past only. A comprehensive search strategy to identify eligible journals will be adopted which is described in more detail below. Afterwards, we will apply specific inclusion criteria for the statistical series on the set of all identified eligible series.

### Information sources & search strategy

With the help of an experienced information retrieval specialist we performed a pilot study with the aim to find sensitive and specific keywords that can identify statistical series within medical journals in bibliographic databases such as MEDLINE. Unfortunately, the pilot study did not reveal common keywords that comprehensively summarize these series. Therefore, we adopted a different search strategy:

We used some known series and started several searches by using potentially relevant keywords. Results from this first search were sent to a group of more than 20 members of the STRATOS collaboration via email in spring 2018. They were asked for additional suggestions on statistical series addressed to medical researchers and we encouraged them to forward this request to their colleagues. We repeated this call at two international STRATOS meetings in summer 2018 and 2019. The search was closed on June 30^st^, 2019. This search strategy therefore resembles snowball sampling, which is a common strategy in sociology to recruit samples from populations that are difficult to reach [16, 17]. Within the email and the call, we also offered the possibility to become a co-author in case of active participation, which can be seen as an incentive to actively support the literature search, so our approach also contains elements of respondent-driven sampling [18].

### Data management & selection process

All statistical series identified by the above search strategy are documented in a list of candidate series including the journal’s name, the journal impact factor, the title of the statistical series (if applicable), the language of the series as well as the overall number of articles in the series (published before 01.01.2019). The list of candidate series is available as S1 File.

For each of the candidate series, two independent raters will check if the series fulfills the following inclusion criteria to be included in the review:

1. The series is published in a medical journal.
2. The series comprises five or more coherent articles.
3. The series is written or translated in English.
4. The series is aimed at an audience with a limited knowledge of statistical education and experience. This includes medical researchers who had some courses in data analysis during their studies but no formal education in statistics. The knowledge of this target audience can be operationalized as being able to understand basic statistical analyses within a publication *and* as performing basic statistical analyses autonomously. (STRATOS level 1).
5. The series contains at least one topic-relevant article.

Within the identified series, both raters will decide independently whether an article can be considered as topic-relevant. In case of disagreement, a consensus must be reached in a discussion among both raters. A series’ article will be considered as topic-relevant if its title includes one of the following keywords (exact, not case sensitive) “Regression”, “Linear”,”Logistic”,”Cox”, “Poisson”,”Multivariable”,”Multivariate”, *or* if it is fairly plausible from the title that the article deals with regression modeling even if the title does not contain one of the above keywords. The rationale behind these keywords is that an applied medical researcher looking for guidance on regression modeling would presumably screen the titles of a series’ articles using those. We do not include the keyword “multiple” as the statistical series include many articles about multiple testing that are not relevant for our research question. Instead, we include the keyword “multivariate” because medical researchers often speak of “multivariate regression”, which relates to a regression model with several dependent variables, when they actually mean a multivariable regression analysis, which relates to a regression model with several independent variables. As the completeness of appropriate keywords is difficult to judge in advance, we also wanted to provide the possibility of including other articles not naming the specific keywords. The rationale is that there might be articles which address a very specific aspect of regression modeling using another wording. As the two independent raters must find an agreement on the topic-relevance of each article, the choice of such articles can still be considered as reasonably reliable.

To screen the series and the articles, we will apply a predefined report form called the “inclusion form” checking the inclusion criteria (S2 and S3 Files). A flow chart will be created to visualize the selection process.

### Data collection process

As soon as agreement on the series to be included and the topic-relevant articles to be screened is reached, a final list containing all identified topic-relevant articles will be created. Each topic-relevant article will be assigned a unique identifier. The two independent raters will then perform a content analysis for all identified topic-relevant articles using our predefined “article content form” (S3 File). This report form will be completed by both raters for every identified topic-relevant article. In case of disagreement, once again a consensus has to be reached in a discussion with both raters. Subsequently, the answers on the article content form will be electronically recorded resulting in the final data set that is used for the analysis to evaluate the research questions.

### Data items

The article content form intends to summarize 44 statistical aspects related to regression modeling of a topic-relevant article in a systematic way. It also allows to collect additional aspects if necessary. The 44 aspects are grouped into the following four statistical areas:

1. The type of regression model,
2. General aspects of regression modeling,
3. Functional form of continuous predictors,
4. Selection of variables.

A comprehensive list of aspects to be considered in this review can be found in the enclosed article content form (S3 File). For each of them we will check whether it is explained at all (yes / no) and we will judge the extent of the explanation (small / medium / large). Furthermore, we will check whether illustrative examples or software commands are provided (yes / no). In addition, we will check whether a particular aspect is explicitly recommended or if a warning against its use is issued. If a recommendation or a warning is provided, the raters are supposed to document the recommendations and warnings in a comments section at the end of the report form. After final data collection, all recommendations and warnings will be discussed among a group of experts, primarily those involved in the STRATOS initiative, with the intention to find a consensus on agreement or disagreement with the recommendation/warning. The comments section also allows to document any further problems or issues which might come up when doing the content analysis.

To ensure an objective completion of the article content form, we provide a corresponding manual for the raters (S4 File).

In our final data set, we will use the following coding for the extent of the explanation provided: 1 = small, 2 = medium, 3 = large. For all binary variables (explained, example, software, recommendation, warning, comment provided) we will use: 1 = yes, 0 = no. The comments will be stored as plain text.

### Outcomes and prioritization

This is an explorative study using methods of descriptive statistics, i.e. frequencies and proportions, to analyze all 44 statistical aspects. Statistical tests are not performed.

### Risk of bias

A potential bias may arise from the fact that the search strategy might deliver incomplete results. Consequently, there might be non-identified series covering important topics on regression modeling which are ignored in this review. However, the risk for this type of bias seems rather low as many experts in regression analysis contributed to the list of eligible series.

Moreover, the 44 aspects listed in the article content form may be criticized to be incomplete or selective. There are certainly several other aspects that could be checked within the article content form and it is difficult up to impossible to find the one and only correct set of aspects. To address the problem of potential incompleteness, the content form allows the reviewers to add no aspects or to provide any additional remarks in the comment box. Therefore, we feel that the risk of neglecting aspects which are described within the articles is low.

### Patient and public involvement

This review does not include patients or the general public.

### Data synthesis

We will provide article-wise- and series-wise analyses, as some topics are covered within a single article and other topics might be addressed by several articles within a series which *together* cover the specific topic. For the latter view point, all identified topic-relevant articles of a statistical series will be pooled and their result will refer to the entire series.

### Quantitative analyses

On an individual article level, for all identified topic-relevant articles, we plan to calculate percentages for each aspect to quantify how often the aspect is explained, how often this explanation is short (up to one sentence), medium (up to one paragraph) or long (more than one paragraph), how often an example or software comment is provided and how often recommendations and warnings are given. Thereby, we particularly intend to identify aspects that are ignored in the literature. The percentages will be visualized using bar charts. The 44 aspects will be grouped by the afore mentioned categories “type of regression model”, “general aspects of regression modeling”, “determination of functional form for continuous predictors” and “selection of variables”.

On a series level, the same analyses will be repeated summarizing all articles among a single series. Thus, an aspect is considered as “explained” if it is explained in at least one of the topic-relevant articles belonging to the series.

### Meta-bias(es) & confidence in cumulative evidence

The quality of evidence provided by this review will be rated according to the severity of potential biases. A potential selection bias may be induced by our search for relevant statistical series in medical journals. Not all statistical series in medical journals may have been identified because we mainly subject our search to the accessibility of series on selected journals’ webpages or to statistical series already known to the aforementioned expert panel. A systematic search is not possible because statistical series cannot be summarized under common keywords. Thus, there certainly lies a potential bias in the fact that the series identified will probably correspond to rather well-established journals. Less prestigious journals might not be known to the experts. This may impose a bias towards higher-quality journals. However, the well-established journals are also the primary focus of interest of the readership. Therefore, this potential bias does not impose a severe problem for the impact of the results.

An aspect that could possibly downgrade the quality of our review is that we might miss to investigate some important aspects of statistical modeling in our report form. However, the case report form was developed and discussed by several experienced researchers working in the field of statistical modeling. The main goal of this review is to identify important general gaps in the knowledge transfer from statistical experts to medical researchers via series on statistical analyses in medical journals, and we are less interested in identifying gaps that refer to very specific aspects that require a high level of statistical knowledge. An overview of important gaps from the latter perspective was recently provided by the corresponding topic group (TG2) of the STRATOS initiative [3].

Another possible criticism is that we only search for statistical series whereas there might be other educational papers on statistical modeling that are published as single articles. However, we believe that the visibility of an entire series and thereby the educational impact is much higher for statistical series than for isolated articles on average. This does not negate that there are excellent isolated articles, which can have a high impact for training medical researchers. Moreover, there exist a variety of introductory textbooks, educational workshops and online video tutorials, some of them with a really good quality. However, as workshops and video tutorials do not have to pass a peer-review process, it might be difficult for the consumer to judge the quality of these offers. For textbooks, the focus is much broader than for articles and therefore a fair comparison is difficult.

In conclusion, we feel that the level of evidence for this specific setting is high. We therefore claim that this protocol will allow to perform a reliable review of available education and guidance on the use of regression analysis in medical research.

### Ethics and dissemination

This protocol has been written according to the PRISMA-P reporting guideline [19, 20], compare S5 File. This review does not include patients or humans. The data that will be collected within the review will be made fully available without restriction upon study completion. Pilot data are available as S5 File.

## Conclusion

Medical researchers with a limited background in statistical methodology need guidance about suitable methods which do not require detailed statistical knowledge and can be easily implemented but which are still acceptable and usable for many analyses. This comprehensive review will provide information to what extent statistical aspects of regression modeling are covered in statistical series within medical journals. The planned quantitative analyses will identify methodological gaps and misconceptions that may have an important impact on the quality of medical research. This review will serve as basis to fill these gaps in the future by developing targeted statistical guidance and tutorials on regression modeling for medical researchers.

## Supporting information

S1 File List of candidate series for potential inclusion in the review. (DOCX)

S2 File Case report form–series inclusion. (DOCX)

S3 File Case report form–article screening. (DOCX)

S4 File Manual for the article screening sheet. (DOCX)

S5 File Check list PRISMA-P reporting guideline. (DOCX)
