# Supplementary material for: Systematic review of education and practical guidance on regression modeling for medical researchers who lack a strong statistical background: Study protocol
Source: PLoS One. 2020 Dec 21;15(12):e0241427. doi: 10.1371/journal.pone.0241427 (PMC7751867; doi:10.1371/journal.pone.0241427)
Supplement: S1 File — (DOCX) [file pone.0241427.s001.docx]

**List of candidate series for potential inclusion in the review**

| ID | Journal | Title of the series |
| --- | --- | --- |
| 1 | The BMJ | Statistics Notes |
| 2 | JAMA | Guide to Statistics and Methods |
| 3 | Deutsche Zahnärztliche Zeitschrift | EbM-Splitter |
| 4 | Deutsches Ärzteblatt | Serie zur Bewertung wissenschaftlicher Publikationen |
| 5 | Deutsches Ärzteblatt International | Series on Evaluation of Scientific Publications |
| 6 | Deutsche Medizinische Wochenzeitschrift | Statistik-Serie in der DMW |
| 7 | Journal of Thoracic Disease | Statistics Corner Column |
| 8 | Advances in Physiology Education | Explorations in statistics column |
| 9 | Archives of Disease in Childhood | Statistics from the inside series |
| 10 | Malawi Medical Journal | Statistics Corner |
| 11 | The Medical Journal of Australia | Accessible series on statistics for clinicians |
| 12 | Circulation | Statistical Primer for Cardiovascular Research |
| 13 | The European Journal of Cardio-Thoracic Surgery & Interactive CardioVascular and Thoracic Surgery journal | Statistics Primers |
| 14 | Radiology | Statistical Concepts Series |
| 15 | Journal of Clinical Oncology | Statistics in Oncology Series |
| 16 | The Annals of Thoracic Surgery | The statistician's page |
| 17 | European Heart Journal | Statistical Tutorials |
| 18 | Kidney International | ABC of Epidemiology |
| 19 | Nephron Clinical Practice | Kidney Disease and population health |
| 20 | Nephrology Dialysis Transplantation | Clinical research in kidney diseases |
| 21 | JAMA | Users' Guide to the Medical Literature |
| 22 | Emergency Medicine Journal | An introduction to statistics |
| 23 | The BMJ | Statistics at Square One |
| 24 | American Journal of Epidemiology | Practice of Epidemiology Issue Section |
| 25 | Statistics in Medicine | Tutorials in Biostatistics Series |
| 26 | Nederlands Tijdschrift van Geneeskunde | ??? |
| 27 | American Physiological Society | Guidelines for reporting statistics in journals |
| 28 | Annals of Allergy, Asthma & Immunology | The reading, writing, and arithmetic of the medical literature |
| 29 | Clinical Radiology | A guide to clinical epidemiology for radiologists: Part I and II |
| 30 | Journal of the American College of Surgeons | Interpreting statistics in medical literature: A vade mecum for surgeons |
| 31 | Journal of Medical Systems | Methodological and Statistical Techniques: What Do Residents Really Need to Know About Statistics? |
| 32 | Postgraduate Medical Journal | Development of the Biostatistics and Clinical Epidemiology Skills |
| 33 | RadioGraphics | Statistics 101 for radiologists |
| 34 | Columbia University | Population Health Methods |
| 35 | Medizinische Monatszeitschrift für Pharmazeuten | Fortbildung Wissensbasierung |
