# Supplementary material for: Systematic review of education and practical guidance on regression modeling for medical researchers who lack a strong statistical background: Study protocol
Source: PLoS One. 2020 Dec 21;15(12):e0241427. doi: 10.1371/journal.pone.0241427 (PMC7751867; doi:10.1371/journal.pone.0241427)
Supplement: S2 File — (DOCX) [file pone.0241427.s002.docx]

| **Journal** |  |
| --- | --- |
| **Journal impact factor (Scopus, 2018)** |  |
| **Statistical series name (if applicable)** |  |
| **Overall number of articles (until 1.1.2019)** |  |

| **Name of rater** |  |
| --- | --- |
| **Date of rating** |  |

| **Inclusion criteria (series)** | Y |  | N |
| --- | --- | --- | --- |
|  |  |  |  |
| 1. Published in a medical journal |  |  |  |
|  |  |  |  |
| 1. Statistical series with 5 or more coherent articles |  |  |  |
|  |  |  |  |
| 1. Written in English |  |  |  |
|  |  |  |  |
| 1. Target audience with limited background in statistics |  |  |  |
|  |  |  |  |
| 1. The series contains at least one topic-relevant article |  |  |  |
|  |  |  |  |
|  |  |  |  |
| **Are all inclusion criteria met?** |  |  |  |

We consider an article as topic-relevant, if:

1. Its title contains one of the key words “regression”, “linear”, “logistic“, “Cox”, “Poisson”, “multivariable” or “multivariate” or
2. It is fairly plausible from the title that the article deals with regression modeling even if the title does not contain one of the above key words.

**Identified topic-relevant articles** *(sort according to year of publication, start with oldest article)*

| **No** | **Rank** | **Title** | **First Author** | **Year of publication** |
| --- | --- | --- | --- | --- |
| 1 |  |  | *First Name, Last Name* |  |
| 2 |  |  |  |  |
| 3 |  |  |  |  |
| 4 |  |  |  |  |
| 5 |  |  |  |  |
| 6 |  |  |  |  |
| 7 |  |  |  |  |
| 8 |  |  |  |  |

*Comments*

| *E.g. None or Does not qualify because it is a single article and not a series.* |
| --- |
