# Supplementary material for: Systematic review of education and practical guidance on regression modeling for medical researchers who lack a strong statistical background: Study protocol
Source: PLoS One. 2020 Dec 21;15(12):e0241427. doi: 10.1371/journal.pone.0241427 (PMC7751867; doi:10.1371/journal.pone.0241427)
Supplement: S3 File — (DOCX) [file pone.0241427.s003.docx]

| **Name of rater** |  |
| --- | --- |
| **Date of rating** |  |

| **Journal** |  |
| --- | --- |
| **Statistical series** |  |
| **Title of the article** |  |
| **Rank of the article within the series** |  |
| **Author(s) of the article** |  |
| **Year of publication of the article** |  |

| **Aspect**  **Number** | | **Explained (y/n)** | **Extent of explanation (S/M/L)** | **Example provided (y/n)** | **Software advice given (y/n)** | **Recommendation given^[[1]](#footnote-1)^ (y/n)** | **Warning issued**  **(y/n)** | **Rater comment^[[2]](#footnote-2)^ (y/n)** |
| --- | --- | --- | --- | --- | --- | --- | --- | --- |
| **1** | **Type of regression model** |  |  |  |  |  |  |  |
| 1.1 | Univariable regression |  |  |  |  |  |  |  |
| 1.2 | Multivariable regression |  |  |  |  |  |  |  |
| 1.3 | Linear regression |  |  |  |  |  |  |  |
| 1.4 | Logistic regression |  |  |  |  |  |  |  |
| 1.5 | Cox regression |  |  |  |  |  |  |  |
| 1.6 | Poisson regression |  |  |  |  |  |  |  |
| 1.7 |  |  |  |  |  |  |  |  |
| 1.8 |  |  |  |  |  |  |  |  |
| **2** | **General aspects of regression modeling** |  |  |  |  |  |  |  |
| 2.1 | Different purposes of regression models |  |  |  |  |  |  |  |
| 2.2 | Interpretation of regression coefficients |  |  |  |  |  |  |  |
| 2.3 | Check of model assumptions |  |  |  |  |  |  |  |
| 2.4 | Correlation coefficient |  |  |  |  |  |  |  |
| 2.5 | Coefficient of determination |  |  |  |  |  |  |  |
| 2.6 | Adjusted coefficient of determination |  |  |  |  |  |  |  |
| 2.7 | Treatment of binary predictors |  |  |  |  |  |  |  |
| 2.8 | Treatment of categorical predictors |  |  |  |  |  |  |  |
| 2.9 | Hypothesis testing for regression coefficients |  |  |  |  |  |  |  |
| 2.10 | Multicollinearity |  |  |  |  |  |  |  |
| 2.11 | Interactions |  |  |  |  |  |  |  |
| 2.12 | Outliers |  |  |  |  |  |  |  |
| 2.13 | Missing values |  |  |  |  |  |  |  |
| 2.14 | Measurement error |  |  |  |  |  |  |  |
| 2.15 | Overfitting |  |  |  |  |  |  |  |
| 2.16 | Number of observations / Events per variable |  |  |  |  |  |  |  |

| **Aspect**  **Number** | | **Explained (y/n)** | **Extent of explanation (S/M/L)** | **Example provided (y/n)** | **Software advice given (y/n)** | **Recommendation given (y/n)** | **Warning issued (y/n)** | **Rater comment (y/n)** |
| --- | --- | --- | --- | --- | --- | --- | --- | --- |
| 2.17 | Visualizing regression results |  |  |  |  |  |  |  |
| 2.18 | Random effect models |  |  |  |  |  |  |  |
| 2.19 | Regression diagnostics |  |  |  |  |  |  |  |
| 2.20 | Model validation |  |  |  |  |  |  |  |
| 2.21 | Reporting regression results |  |  |  |  |  |  |  |
| 2.22 |  |  |  |  |  |  |  |  |
| 2.23 |  |  |  |  |  |  |  |  |
| **3** | **Functional form of continuous predictors** |  |  |  |  |  |  |  |
| 3.1 | Possibility of a nonlinear relation |  |  |  |  |  |  |  |
| 3.2 | Dichotomization of continuous predictors |  |  |  |  |  |  |  |
| 3.3 | Nonlinear transformations |  |  |  |  |  |  |  |
| 3.4 | Polynomial regression |  |  |  |  |  |  |  |
| 3.5 | Fractional polynomials |  |  |  |  |  |  |  |
| 3.6 | Splines |  |  |  |  |  |  |  |
| 3.7 | Generalized additive models |  |  |  |  |  |  |  |
| 3.8 |  |  |  |  |  |  |  |  |
| 3.9 |  |  |  |  |  |  |  |  |
| **4** | **Selection of variables** |  |  |  |  |  |  |  |
| 4.1 | Selection by background knowledge |  |  |  |  |  |  |  |
| 4.2 | Univariate screening |  |  |  |  |  |  |  |
| 4.3 | Forward Selection |  |  |  |  |  |  |  |
| 4.4 | Backward Elimination |  |  |  |  |  |  |  |
| 4.5 | Stepwise Selection |  |  |  |  |  |  |  |
| 4.6 | Choice of the „significance level“ |  |  |  |  |  |  |  |
| 4.7 | Selection by AIC/BIC |  |  |  |  |  |  |  |
| 4.8 | Selection by Lasso |  |  |  |  |  |  |  |
| 4.9 | Instability of data-driven selection |  |  |  |  |  |  |  |
| 4.10 | Post-selection inference |  |  |  |  |  |  |  |
| 4.11 |  |  |  |  |  |  |  |  |
| 4.12 |  |  |  |  |  |  |  |  |

*Comments*

| Refers to: Please specify Aspect Number/ General comment |
| --- |

1. Please write down any recommendation or warning in the comment section at the end of the sheet. [↑](#footnote-ref-1)
2. Please indicate if you have a comment on the explanation of a particular aspect. [↑](#footnote-ref-2)
